# Rapid and sustained improvements in health-related quality of life, fatigue, and other patient-reported outcomes in rheumatoid arthritis patients treated with certolizumab pegol plus methotrexate over 1 year: results from the RAPID 1 randomized controlled trial

**DOI:** 10.1186/ar2859

**Published:** 2009-11-12

**Authors:** Vibeke Strand, Philip Mease, Gerd R Burmester, Enkeleida Nikaï, Geoffroy Coteur, Ronald van Vollenhoven, Bernard Combe, Edward C Keystone, Arthur Kavanaugh

**Affiliations:** 1 Division of Immunology/Rheumatology, Stanford University School of Medicine, 306 Ramona Road, Palo Alto, CA, 94028, USA; 2 Seattle Rheumatology Associates, 1101 Madison St # 1000, Seattle, WA, 98104, USA; 3 Department of Rheumatology and Clinical Immunology, Charité - University Medicine, Humboldt University of Berlin, Unter den Linden 6, Berlin, D-10099, Germany; 4 Life Sciences, Business & Decision, Rue Saint Lambert 141, 1200 Brussels, Belgium; 5 Outcomes & Access - Immunology, UCB SA, Allée de la Recherche 60, B-1070, Brussels, Belgium; 6 Rheumatology Unit, Karolinska Institute, SE-171 77, Stockholm, Sweden; 7 Immuno-Rhumatologie, Hôpital Lapeyronie, Université Montpellier 1, Montpellier, F-34000, France; 8 Rebecca MacDonald Centre for Arthritis & Autoimmune Disease, Mount Sinai Hospital, 60 Murray St., Room 2-006, Box 4, Toronto, Ontario, M5T 3L9, Canada; 9 Division of Rheumatology, Allergy and Immunology, University of California in San Diego, 9500 Gilman Drive, Mail code 0943, La Jolla, CA, 92093, USA

## Abstract

**Introduction:**

The objective of this study was to assess the impact of certolizumab pegol (CZP) treatment on health-related quality of life (HRQoL), fatigue and other patient-reported outcomes (PROs) in patients with rheumatoid arthritis (RA).

**Methods:**

Patients with active RA (N = 982) were randomized 2:2:1 to subcutaneous CZP (400 mg at weeks 0, 2 and 4; followed by CZP 200 mg or 400 mg) plus methotrexate (MTX) every other week, or placebo (PBO) plus MTX. PRO assessments included HRQoL, fatigue, physical function, arthritis pain and disease activity. Adjusted mean changes from baseline in all PROs were obtained using analysis of covariance (ANCOVA) applying last observation carried forward (LOCF) imputation. The proportion of patients achieving clinically meaningful improvements in each PRO was obtained using logistic regression and by applying non-responder imputation to missing values after rescue medication or withdrawal. The correlations between PRO responses and clinical responses were also assessed by tetrachoric correlation using non-responder imputation.

**Results:**

Patients treated with CZP plus MTX reported significant (*P *< 0.001), clinically meaningful improvements in HRQoL at the first assessment (week 12); reductions in fatigue, disease activity and pain and improvements in physical function were reported at week 1. In particular, CZP-treated patients reported improvements in mental health. Mean changes from baseline in the SF-36 Mental Component Summary (MCS) at week 52 for CZP 200 mg and 400 mg plus MTX, and PBO plus MTX were 6.4, 6.4 and 2.1, respectively (*P *< 0.001). In addition, mental health and vitality scores in CZP-treated patients approached age- and gender-adjusted US population norms. Improvements in all PROs were sustained. Similar benefits were reported with both CZP doses. Changes in SF-36 MCS scores had the lowest correlation with disease activity scores (DAS28) and American College of Rheumatology 20% improvement (ACR20) response rates, while improvements in pain showed the highest correlation.

**Conclusions:**

Treatment with CZP plus MTX resulted in rapid and sustained improvements in all PROs, indicating that the benefits of CZP extend beyond clinical efficacy endpoints into areas that are more relevant and meaningful for patients on a daily basis.

**Trial Registration:**

ClinicalTrials.gov NCT00152386.

## Introduction

Rheumatoid arthritis (RA) is a common severe inflammatory disorder characterized by progressive joint damage and functional impairments [1]. It has been widely reported that the daily-life burdens associated with RA, including functional impairment, chronic and debilitating pain, inability to participate in desired family, social and leisure activities and reduced productivity at work and within the home, have a profound impact on an individual's health-related quality of life (HRQoL) [2-5]. As such, HRQoL is now considered to be an essential outcome measure in many clinical studies [6] and the American College of Rheumatology (ACR), the European League Against Rheumatism (EULAR) and the Outcomes Measures in Rheumatology (OMERACT) have recognized the importance of measuring functioning and well-being from the patient's perspective in clinical trials [7].

Another multidimensional burden experienced by almost all RA patients is fatigue. RA-related fatigue has been reported to be more extreme than normal tiredness, to restrict patients' abilities to fulfill their normal family roles and to take a severe emotional toll on patients [8]. Furthermore, an examination of both the physical and mental components of fatigue revealed that high levels of mental fatigue coincide with elevated levels of bodily pain and physical limitations in patients with RA [9].

Assessing patient's burden is an important component in monitoring both the progression of disease and the effectiveness of RA therapies. Physician-reported measures offer the physician's assessment of patient's health, while patient-reported assessments of both the physical (fatigue and pain) and mental burden of RA reflect the impact of disease on everyday life. Moreover, some of these symptoms (especially those that are mental/emotional in nature) are known only to, and can thus only be reported by, patients. An analysis of randomized controlled trials has shown that patient-reported outcomes demonstrate better discrimination of the treatment effect than more traditional physician-reported outcomes [10,11], and are, therefore, the most sensitive tools for assessing the impact of therapy on RA symptoms [12]. Taken together, the patient and physician-reported assessments are complementary and provide a holistic picture of a patient's disease state or well-being.

The efficacy and safety of certolizumab pegol (CZP), the only PEGylated anti-TNF for the treatment of RA, has been established in several phase III clinical trials [13-15]. Previously-reported clinical results from the RA PreventIon of Structural Damage 1 (RAPID 1) clinical trial have demonstrated that CZP, dosed at 200 mg or 400 mg every other week plus methotrexate (MTX), provides rapid reductions in the signs and symptoms of active RA (as assessed by ACR responder rates) and improvements in disease activity (as assessed by disease activity scores [DAS28]) in MTX inadequate responders [15]. In this paper, we present the patient-reported outcome (PRO) results from the RAPID 1 trial, including HRQoL, fatigue, physical function, pain and patient's global assessment of disease activity. To further explore the relation between the patient-reported and clinical (physician-reported) assessments, we also examined the correlations between the PROs and the ACR20 and DAS responses.

## Materials and methods

### Patients

Full details regarding patient inclusion and exclusion criteria and randomization for RAPID 1 were previously published [15]. In brief, patients were aged 18 years or older with active RA (according to the 1987 ACR RA classification criteria [16]) with an inadequate response to MTX therapy (≥ 10 mg weekly for ≥ 6 months with stable doses for ≥ 2 months prior to baseline). Patients were ineligible if they had previously failed to respond to treatment with a TNF inhibitor. All patients provided written informed consent.

### Study design

RAPID 1 was a phase III, double-blind, randomized, placebo (PBO)-controlled, multicenter trial in which patients with active RA were randomized 2:2:1 to receive subcutaneous CZP (400 mg weeks 0, 2 and 4 followed by CZP 200 mg or 400 mg) plus MTX every other week, or PBO plus MTX. Patients not achieving ACR20 responses at weeks 12 and 14 were to be withdrawn from treatment per protocol at week 16. These patients, and those who completed RAPID 1, were eligible to enter an ongoing open-label extension study of CZP 400 mg plus MTX every other week. The study (NCT00152386) was conducted in accordance with the International Conference on Harmonisation E6 Note for Guidance on Good Clinical Practice (CPMP/ICH/135/95) and Declaration of Helsinki. All elements of the CONSORT checklist for reporting randomized clinical trials have been previously published in the primary RAPID 1 publication [15]. Institutional review boards or ethics committees approved the protocol at each center, and all patients provided written consent.

### Patient-reported outcomes

All patient-reported outcomes assessed in the RAPID 1 trial were secondary endpoints. These PROs included evaluations of concepts such as HRQoL, fatigue and the patient-reported components of the ACR core set criteria (physical function, arthritis pain and patient's global assessment of disease activity). The PRO instruments assessed in RAPID 1 have been shown to provide valid and reliable data in prior RA research. They were properly implemented in the CZP trials in terms of patient and investigator training, timing of assessments, linguistic validations, and pre-specification of analysis in the statistical analysis plan. Psychometric properties of the data based on the endpoints of the patient-reported outcomes are consistent with prior evidence of the measurement properties for each instrument.

HRQoL was assessed using the Short-Form 36-Items (SF-36) health survey (Version 1.0 standard recall), which is a widely used generic HRQoL instrument with numerous studies documenting its validity and reliability as an accurate measure of generic health concepts in RA [4,10,17-21]. SF-36 assesses eight domains: Physical Functioning, Role Physical, Bodily Pain, General Health, Vitality, Social Functioning, Role Emotional and Mental Health, scored from 0 to 100, with higher scores indicating better HRQoL [22]. The Physical Component Summary (PCS) and the Mental Component Summary (MCS) scores were obtained from normalized and z-transformed domain scores, with normative values of 50 and standard deviations of 10. Minimum clinically important differences (MCIDs) for the SF-36 domains are defined as a 5.0 or more point increase from baseline and a 2.5 or more point increase from baseline for PCS and MCS scores [23]. Baseline SF-36 domain scores were compared with US population norms adjusted for the age and gender distribution of the trial population.

Fatigue (weariness, tiredness) over the past week was evaluated using the Fatigue Assessment Scale (FAS), a numeric rating scale (NRS) from 0 to 10 with higher scores indicating greater fatigue [24]. The MCID for the FAS is 10% of the scale range, corresponding to a one-point change [25]. Physical function was assessed by the Health Assessment Questionnaire-Disability Index (HAQ-DI), evaluating eight activities of daily living [26,27]. HAQ-DI scores range from 0 to 3, with higher scores indicating lower levels of physical functioning. Improvements of 0.22 or more points from baseline represent the HAQ-DI MCID [28]. Patient's arthritis pain and patient's global assessment of disease activity (PtGA) were evaluated using 0 to 100 mm visual analog scales (VAS), where higher scores indicate greater pain and disease activity. MCIDs for both pain VAS and PtGA are defined as 10 mm improvements from baseline [25,28-30].

The SF-36 was completed at baseline, week 12 and every 12 weeks thereafter until week 52 or withdrawal. All other patient-reported outcomes were assessed at baseline, weeks 1 and 2 and every 2 weeks until week 20, followed by every 4 weeks until week 52 or early withdrawal. Fatigue (FAS) was additionally assessed at weeks 5 and 9.

### Statistical analyses

Analyses were conducted on the intent-to-treat (ITT) population, which included all randomized patients receiving at least 1 dose of study treatment. Changes from baseline in PRO scores were analyzed using Analysis of Covariance (ANCOVA) with region and treatment as factors, and baseline score as covariate (using last observation carried forward (LOCF) imputation of missing data). Sensitivity analyses were performed using the repeated measures direct likelihood method. The proportions of patients reporting improvements equal to or greater than the determined MCID for each PRO (*post hoc *analyses) were compared using repeated-measures logistic regression with region, treatment, time and treatment by time interaction as factors and baseline score as covariate. Response rates (i.e., proportions of patients with clinically meaningful improvements) in pain VAS, HAQ-DI, fatigue NRS, and SF-36 PCS and MCS were compared at week 52 to clinical response rates, defined as a decrease from baseline in DAS28 of 1.2 units or an ACR20 response, by cross tabulation. For analysis of response rates, patients with missing data after rescue medication intake or withdrawal were imputed with a conservative approach of non-responders.

## Results

### Patients

A total of 982 patients were randomized in RAPID 1: 393 to CZP 200 mg plus MTX, 390 to CZP 400 mg plus MTX, and 199 to PBO plus MTX. Of these, 255 (64.9%), 274 (70.3%) and 43 (21.6%) completed 52 weeks of treatment, respectively [28]. At baseline, 82% of patients were female, with mean age 52 years, 77% rheumatoid factor positive, mean disease duration 6.2 years, and having failed a mean of 1.4 disease-modifying antirheumatic drugs (DMARDs; excluding MTX). The burden of RA at baseline was significant, as evidenced by patient-reported global assessment of disease activity, pain, physical function, and fatigue scores (Table 1). In particular, SF-36 domain scores at baseline were markedly lower than age- and gender-matched US population norms, and SF-36 MCS and PCS scores reflected a significant psychological burden as well as physical impairments.

**Table 1:** Baseline disease activity and PRO scores (ITT population)

Characteristic, mean (SD)	CZP 200 mg + MTX (n = 393)	CZP 400 mg + MTX (n = 390)	PBO + MTX (n = 199)
DAS28, range 0-10	6.9 (0.8)	6.9 (0.8)	7.0 (0.9)
PtGA VAS, range 0-100 mm	63.1 (20.3)	64.1 (18.3)	64.2 (19.6)
Pain VAS, range 0-100 mm	62.1 (20.0)	63.8 (17.2)	63.6 (19.9)
HAQ-DI, range 0-3	1.7 (0.6)	1.7 (0.6)	1.7 (0.6)
Fatigue NRS, range 0-10	6.4 (2.0)	6.5 (1.9)	6.7 (2.0)
SF-36			
PCS, range 0-100*	30.9 (6.5)	30.8 (6.8)	30.5 (5.8)
MCS, range 0-100*	40.0 (11.2)	39.3 (11.1)	38.6 (11.4)
Domains, range 0-100			
Physical Functioning	33.4 (21.4)	32.9 (21.2)	32.0 (20.1)
Role Physical	13.3 (23.7)	12.9 (25.4)	11.2 (20.8)
Bodily Pain	30.6 (15.3)	28.9 (15.9)	28.5 (14.7)
General Health	35.3 (16.5)	35.9 (17.5)	34.9 (15.6)
Vitality	35.8 (18.0)	36.1 (18.6)	32.9 (17.4)
Social Functioning	50.9 (23.8)	48.1 (24.0)	46.6 (25.2)
Role Emotional	32.4 (39.1)	28.9 (38.1)	30.9 (38.7)
Mental Health	53.8 (20.1)	53.5 (20.7)	52.2 (21.2)

* mean US population normative value equals 50

CZP = certolizumab pegol; DAS28 = disease activity score; HAQ-DI = health assessment questionnaire-disability index; ITT = intent to treat; MCS = mental component summary; MTX = methotrexate; NRS = numeric rating scale; PBO = placebo; PCS = physical component summary; PRO = patient-reported outcomes; PtGA = patient's global assessment of disease activity; SD = standard deviation; SF-36 = short-form 36-item health survey; VAS = visual analog scale.

### Health-related quality of life and fatigue

**Figure 1:**
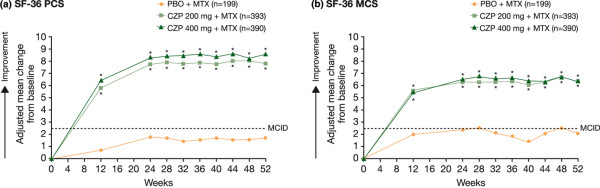
Adjusted mean change from baseline in SF-36 PCS **(a)** and MCS **(b)** scores over 52 weeks (ITT population, LOCF). *P < 0.001 for CZP vs PBO by ANCOVA (LOCF imputation). ANCOVA = analysis of covariance; CZP = certolizumab pegol; ITT = intent to treat; LOCF = last observation carried forward; MCID = minimal clinically important difference; MCS = mental component summary; MTX = methotrexate; PBO = placebo; PCS = physical component summary; SF-36 = short-form 36-item health survey.

RA patients receiving CZP plus MTX reported statistically significant improvements in HRQoL compared with PBO plus MTX by the first post-baseline assessment (week 12; *P *< 0.001); improvements in all eight SF-36 domains (Table 2), PCS (Figure 1a), and MCS (Figure 1b) were sustained to week 52. In particular, mean changes from baseline at week 12 for the CZP 200 mg plus MTX, CZP 400 mg plus MTX, and PBO plus MTX groups were 5.6, 5.5 and 2.0 for the SF-36 MCS, respectively (Table 2; *P *< 0.001). Significantly more patients treated with CZP plus MTX than PBO plus MTX also reported improvements in SF-36 MCS equal to or greater than the MCID from weeks 12 to 52 (Table 2; *P *< 0.05).

**Table 2:** Improvements in health-related quality of life at weeks 12, 24 and 52 (ITT population, LOCF)*

	Week 12			Week 24			Week 52		
			
	CZP 200 mg + MTX (n = 393)	CZP 400 mg + MTX (n = 390)	PBO + MTX (n = 199)	CZP 200 mg + MTX (n = 393)	CZP 400 mg + MTX (n = 390)	PBO + MTX (n = 199)	CZP 200 mg + MTX (n = 393)	CZP 400 mg + MTX (n = 390)	PBO + MTX (n = 199)
PCS									
Mean score (SD)	36.1 (8.9)	36.8 (8.4)	30.7 (7.1)	37.8 (9.5)	38.4 (8.8)	31.4 (7.3)	38.1 (9.5)	38.9 (8.9)	31.5 (7.4)
Mean change from BL	5.8 (0.5)†	6.4 (0.6)†	0.7 (0.6)	7.7 (0.4)†	8.3 (0.4)†	1.8 (0.6)	7.8 (0.4)†	8.6 (0.4)†	1.7 (0.6)
% MCID	38.2†	36.5†	21.4	46.6†	51.7†	10.9	42.2†	46.1†	11.5
MCS									
Mean score (SD)	45.5 (11.5)	45.7 (11.6)	41.4 (10.8)	45.6 (11.5)	45.6 (11.6)	41.3 (10.7)	45.8 (11.4)	45.4 (11.5)	41.1 (10.8)
Mean change from BL	5.6 (0.7)†	5.5 (0.7)†	2.0 (0.8)	6.3 (0.6)†	6.5 (0.6)†	2.3 (0.8)	6.4 (0.6)†	6.4 (0.6)†	2.1 (0.8)
% MCID	36.0‡	33.3	28.1	41.1†	41.9†	13.0	39.2†	38.1†	9.9
Physical Functioning									
Mean score (SD)	45.1 (25.8)	46.1 (24.6)	31.2 (21.0)	47.6 (26.5)	48.8 (24.7)	33.3 (22.2)	49.3 (26.9)	50.3 (24.9)	33.1 (22.3)
Mean change from BL	11.4 (1.4)†	12.2 (1.4)†	-1.0 (1.7)	15.6 (1.2)†	16.5 (1.2)†	1.9 (1.6)	16.7 (1.2)†	17.9 (1.2)†	1.7 (1.6)
% MCID	32.1†	33.6†	18.4	42.0†	46.6†	10.7	38.0†	44.3†	9.7
Role Physical									
Mean score (SD)	34.1 (38.3)	33.9 (38.5)	15.0 (28.0)	37.2 (40.4)	38.6 (40.2)	18.0 (30.2)	37.7 (40.3)	39.2 (41.8)	17.3 (30.2)
Mean change from BL	24.8 (2.4)†	24.2 (2.5)†	6.4 (2.9)	27.8 (2.0)†	29.1 (2.0)†	9.4 (2.8)	26.9 (2.1)†	29.1 (2.0)†	8.1 (2.9)
% MCID	24.9†	24.5‡	13.9	33.0†	36.8†	10.3	32.2†	31.1†	8.2
Bodily Pain									
Mean score (SD)	47.8 (20.1)	49.6 (20.4)	33.1 (18.5)	51.4 (22.3)	53.4 (20.8)	33.2 (19.4)	52.0 (22.8)	53.9 (22.2)	34.0 (20.1)
Mean change from BL	18.0 (1.3)†	20.2 (1.4)†	4.5 (1.6)	23.3 (1.1)†	25.8 (1.1)†	6.3 (1.5)	23.5 (1.1)†	26.2 (1.1)†	6.8 (1.6)
% MCID	45.9‡	43.3‡	33.3	52.5†	58.2†	15.9	48.8†	50.7†	14.9
General Health									
Mean score (SD)	44.0 (18.9)	48.4 (19.6)	36.9 (18.0)	47.4 (19.6)	48.5 (19.7)	37.2 (17.1)	47.5 (20.3)	47.6 (19.2)	36.9 (16.6)
Mean change from BL	8.9 (1.1)†	11.1 (1.1)†	2.4 (1.3)	13.2 (0.9)†	14.0 (0.9)†	3.7 (1.2)	13.0 (0.9)†	13.0 (0.9)†	3.1 (1.3)
% MCID	31.1	32.7‡	24.2	44.3†	45.7†	11.3	40.3†	40.6†	10.3
Vitality									
Mean score (SD)	48.2 (21.4)	50.0 (21.3)	37.0 (19.1)	50.9 (21.6)	51.4 (21.3)	38.3 (19.9)	50.7 (21.7)	51.1 (20.9)	38.4 (20.0)
Mean change from BL	12.9 (1.3)†	14.4 (1.4)†	2.8 (1.6)	15.5 (1.1)†	16.2 (1.1)†	4.7 (1.5)	15.1 (1.0)†	15.6 (1.0)†	4.5 (1.5)
% MCID	35.5‡	35.3‡	27.0	45.1†	45.7†	12.2	40.8†	42.8†	11.2
Social Functioning									
Mean score (SD)	64.8 (25.2)	64.0 (24.4)	50.6 (25.1)	66.6 (25.7)	65.7 (24.4)	50.8 (25.7)	67.2 (25.9)	66.2 (25.3)	50.6 (25.4)
Mean change from BL	15.6 (1.6)†	14.7 (1.6)†	2.6 (1.9)	18.3 (1.3)†	18.1 (1.3)†	3.4 (1.8)	18.5 (1.3)†	18.6 (1.3)†	3.2 (1.8)
% MCID	39.5‡	36.8	30.1	45.3†	48.3†	11.7	43.5†	44.4†	11.2
Role Emotional									
Mean score (SD)	50.4 (43.0)	50.8 (43.9)	37.4 (41.2)	50.2 (42.9)	54.1 (43.4)	36.3 (40.9)	52.8 (43.0)	53.9 (43.5)	35.7 (40.6)
Mean change from BL	19.2 (2.8)‡	19.3 (2.9)‡	6.6 (3.3)	22.2 (2.2)†	26.4 (2.2)†	7.8 (3.1)	23.9 (2.3)†	26.1 (2.2)†	7.1 (3.1)
% MCID	25.5‡	22.4	17.4	31.7†	33.2†	10.8	29.0†	29.7†	9.2
Mental Health									
Mean score (SD)	64.1 (19.4)	64.5 (20.2)	56.1 (19.7)	64.3 (20.1)	63.8 (20.2)	56.3 (21.1)	64.3 (20.4)	63.3 (19.9)	55.8 (21.1)
Mean change from BL	9.3 (1.2)†	8.9 (1.2)†	2.1 (1.4)	11.0 (1.0)†	10.5 (1.0)†	3.7 (1.3)	10.7 (1.0)†	9.9 (1.0)†	3.0 (1.4)
% MCID	33.3‡	27.4	27.0	40.8†	37.1†	14.3	37.3†	33.9†	11.2

*SF-36 PCS, MCS and domain scores, adjusted mean change from baseline, and percentage of patients reporting improvements meeting or exceeding MCID. Changes from baseline were analyzed using ANCOVA with region and treatment as factors, and baseline as covariate (using LOCF imputation of missing data).

†*P *< 0.001; ‡*P *< 0.05.

BL = baseline; CZP = certolizumab pegol; ITT = intent to treat; LOCF = last observation carried forward; MCID = minimal clinically important difference; MCS = mental component summary; MTX = methotrexate; PBO = placebo; PCS = physical component summary; SD = standard deviation; SF-36 = short-form 36-item health survey.

**Figure 2:**
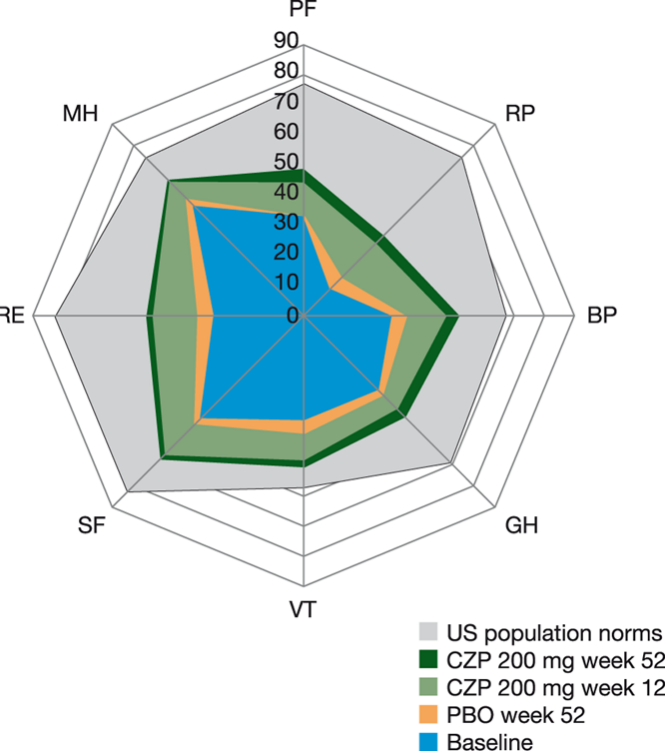
Spydergram of SF-36 domains at baseline and weeks 12 and 52 (ITT population, LOCF). Physical domains: Physical Function (PF), Role Physical (RP), Bodily Pain (BP), General Health (GH). Mental domains: Vitality (VT), Social Function (SF), Role Emotional (RE), Mental Health (MH). Domain scores are plotted from 0 (worst) at the center to 100 (best) at the outside; demarcations along axes of the domains present changes of 10 points, representing 1 - 2 times MCID. As the RAPID 1 protocol included RA subjects recruited outside North America, available US normative data offer a 'benchmark', but less realistic goal for therapy. Changes from baseline for all SF-36 domains were statistically significant for CZP versus PBO at Weeks 12 and 52; *P *< 0.05. CZP = certolizumab pegol; ITT = intent to treat; LOCF = last observation carried forward; MCID = minimal clinically important difference; PBO = placebo; RA = rheumatoid arthritis; RAPID 1 = RA PreventIon of Structural Damage 1; SF-36 = short-form 36-item health survey.

Figure 2 shows the SF-36 domain scores for the CZP 200 mg and PBO groups compared with normative values from an age/gender-matched US population. Baseline domain scores reflect the largest decrements in the Role Emotional mental domain, and the physical domains of Role Physical and Physical Function (Figure 2). Improvements exceeding MCID were evident in all domains at week 12 and were sustained through week 52, with the Mental Health and Vitality domains approaching US population normative values.

**Figure 3:**
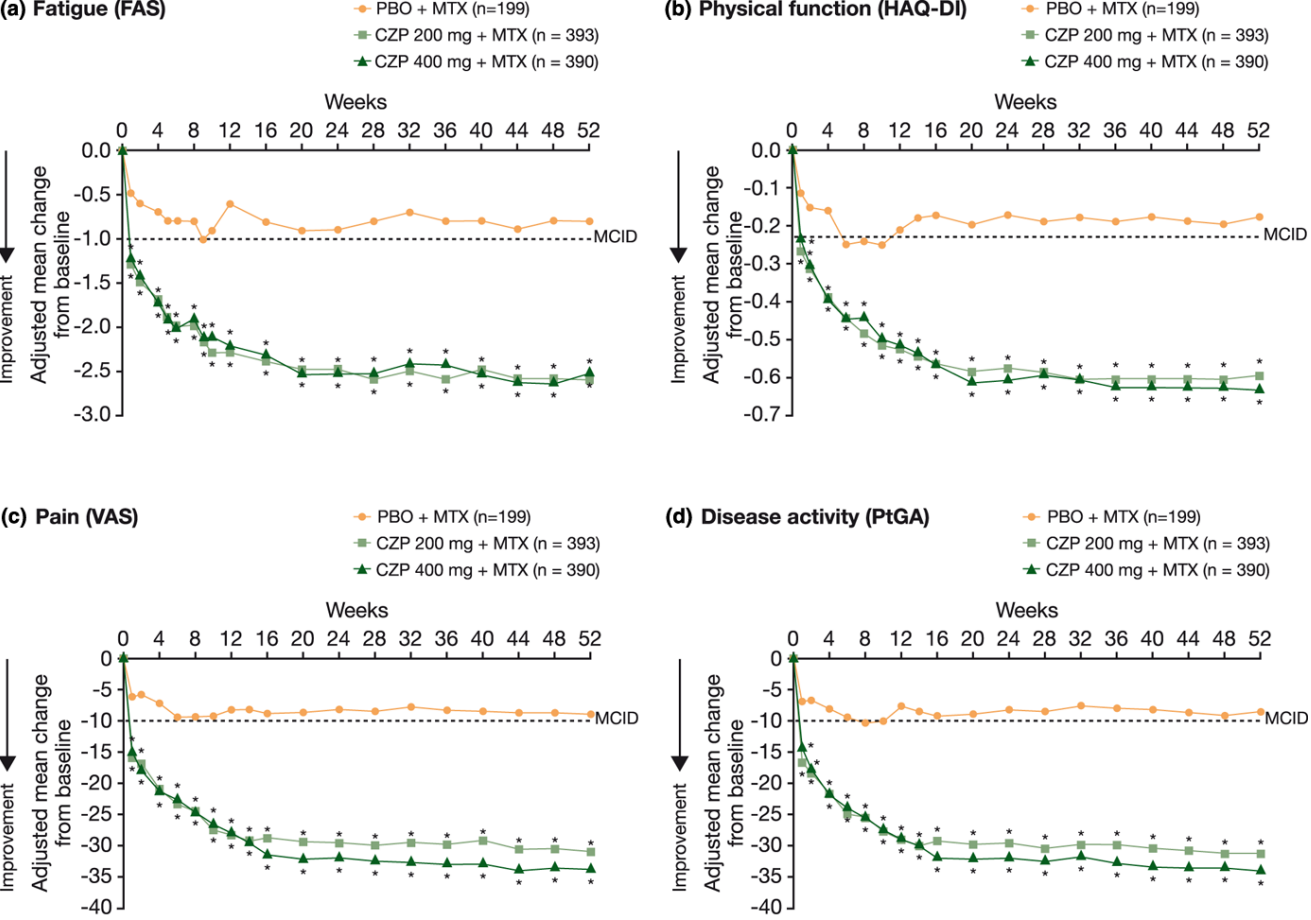
Improvements in fatigue **(a)**, physical function **(b)**, pain **(c)** and disease activity **(d)** over 52 weeks (ITT population, LOCF). *P < 0.001 for CZP vs PBO by ANCOVA (LOCF imputation). ANCOVA = analysis of covariance; CZP = certolizumab pegol; FAS = fatigue assessment scale; HAQ-DI = health assessment questionnaire - disability index; ITT = intent to treat; LOCF = last observation carried forward; MCID = minimal clinically important difference; MTX = methotrexate; PBO = placebo; PtGA = patient's global assessment of disease activity; VAS = visual analog scale.

**Figure 4:**
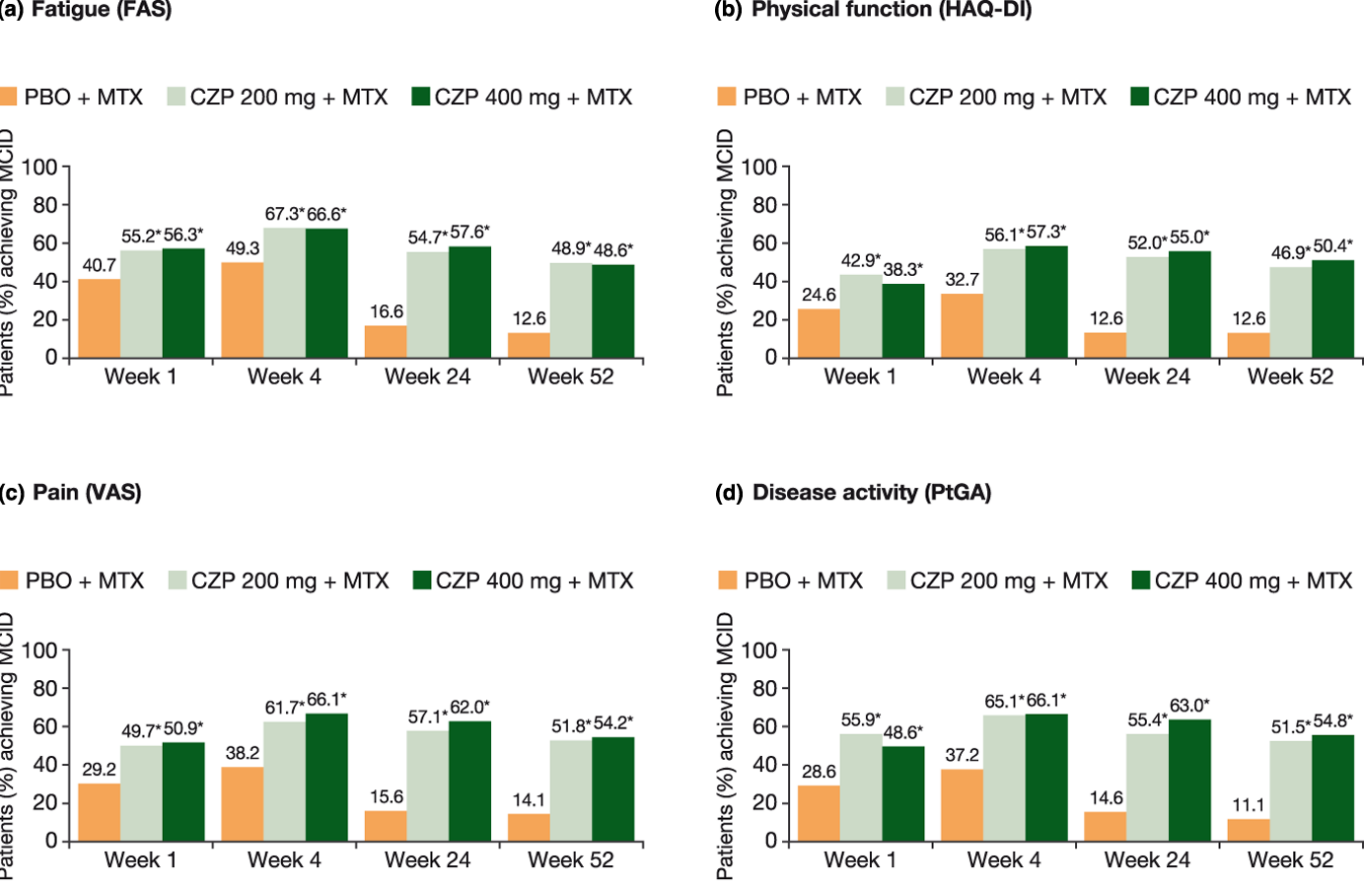
Patients (%) achieving minimum clinically important differences in fatigue **(a)**, physical function **(b)**, pain **(c)**, and disease activity **(d)** over 52 weeks (ITT population, LOCF). *P < 0.001 for CZP vs PBO by repeated measures logistic regression. CZP = certolizumab pegol; FAS = fatigue assessment scale; HAQ-DI = health assessment questionnaire - disability index; ITT = intent to treat; LOCF = last observation carried forward; MCID = minimal clinically important difference; MTX = methotrexate; PBO = placebo; PtGA = patient's global assessment of disease activity; VAS = visual analog scale.

Statistically significant and clinically meaningful reductions in fatigue were reported by more patients treated with CZP plus MTX than PBO plus MTX throughout the study (*P *< 0.001; Figure 3a). At week 1, mean changes from baseline in FAS were -1.3 and -1.2 for CZP 200 mg and 400 mg plus MTX, respectively, compared with -0.5 for the PBO plus MTX group (*P *< 0.001), and by the end of the study (week 52), mean changes from baseline were -2.6, -2.5 and -0.8, respectively (*P *< 0.001). Statistically more CZP plus MTX patients also reported reductions in fatigue equal to or greater than the MCID compared with PBO plus MTX (*P *< 0.001; Figure 4a). At week 52, 48.9% and 48.6% of CZP 200 mg- and 400 mg-treated patients reported fatigue reductions equal to or greater than the MCID compared with only 12.6% of PBO-treated patients (*P *< 0.001).

### Patient-reported physical function, pain and global assessment of disease activity

Rapid, statistically significant improvements in physical function, as assessed by HAQ-DI, were reported by patients treated with CZP plus MTX compared with PBO plus MTX (Figure 3b; *P *< 0.001). By the end of the study at week 52, mean scores were 1.1 for both CZP plus MTX treatment groups, respectively, compared with baseline scores of 1.7. Significantly more CZP plus MTX-treated patients than PBO plus MTX-treated patients reported HAQ-DI improvements equal to or greater than the MCID throughout the trial (*P *< 0.001). CZP-treated patients also reported statistically significant improvements in physical function that met or exceeded MCID as reported above for SF-36 PCS (Figure 1a) and Physical Functioning domain scores (Table 2).

CZP-treated patients reported significant improvements from baseline in pain VAS (Figure 3c) and patient's global assessment of disease activity (Figure 3d; *P *< 0.001), evident at week 1 and sustained through week 52. Significantly more CZP plus MTX-treated patients than PBO plus MTX-treated patients also reported clinically meaningful reductions in pain (Figure 4c) and improvements in global assessment of disease activity (Figure 4d) throughout the trial (*P *< 0.001). Results were similar regardless of CZP dose (200 mg or 400 mg).

Sensitivity analyses showed that improvements in all PROs were consistent with the LOCF results.

### Comparison of patient-reported outcomes with DAS28 and ACR20 response rates

To explore the relation between the patient-reported outcomes and the physician-reported assessments of disease, PRO responses (as defined by improvements in physical function, pain, fatigue and SF-36 scores equal to or greater than the MCIDs) were compared with DAS28 responses (defined as a decrease from baseline of ≥ 1.2) and ACR20 response rates at week 52. In general, PRO responses were in agreement with clinical responses as determined by either improvements in RA signs and symptoms (ACR20 response rates) or decrease in disease activity (DAS28 scores; Table 3). As expected, classification of response according to physical function and pain was highly correlated with ACR20 response rates, with 88.8% and 93.6% of patients treated with CZP 200 mg plus MTX being classified the same by physical function/ACR20 and pain/ACR20, respectively. Correlations between fatigue and ACR20 responses were also high (90.0%) as were those between patient's global assessment of disease activity (98.0%), while changes in HRQoL (SF-36 PCS and MCS scores) had the lowest correlation with ACR20 responses (84.3% and 77.4%, respectively).

**Table 3:** PRO response by clinical response* at week 52 (CZP 200 mg plus MTX group, ITT population)

		ACR20 response by (%)											
		
		SF-36 PCS		SF-36 MCS		HAQ-DI		Pain		FAS		PtGA	
	**Clinical response**	No	Yes	No	Yes	No	Yes	No	Yes	No	Yes	No	Yes
**PRO response**	No	45.5	14.7	43.4	19.5	44.4	8.7	44.4	3.8	44.1	7.1	86.9	2.0
	Yes	1.0	38.8	3.1	33.9	2.6	44.4	1.0	38.2	2.8	45.9	0	11.1
	Tetrachoric correlation (ASE)	0.9450 (0.0178)		0.8389 (0.0356)		0.9504 (0.0152)		0.9804 (0.0076)		0.9574 (0.0135)		0.9996 (0.0053)	
													
		**DAS response by (%)**											
		
		**SF-36 PCS**		**SF-36 MCS**		**HAQ-DI**		**Pain**		**FAS**		**PtGA**	

	**Clinical response**	No	Yes	No	Yes	No	Yes	No	Yes	No	Yes	No	Yes
**PRO response**	No	44.2	16.5	43.2	20.4	43.6	10.0	43.6	5.1	43.9	7.7	44.1	4.9
	Yes	1.0	38.2	2.1	34.4	2.1	44.4	2.1	49.2	1.8	46.7	1.5	49.5
	Tetrachoric correlation (ASE)	0.9344 (0.0204)		0.8674 (0.0322)		0.9496 (0.0156)		0.9776 (0.0085)		0.9672 (0.0114)		0.9833 (0.0069)	

*Clinical response = DAS or ACR20 response.

ACR20 = American College of Rheumatology 20% improvement; ASE = asymptomatic standard error; CZP = certolizumab pegol; DAS = disease activity score; FAS = fatigue assessment scale; HAQ-DI = health assessment questionnaire-disability index; ITT = intent to treat; MCS = mental component summary; MTX = methotrexate; PtGA = patient's global assessment; PCS = physical component summary; PRO = patient-reported outcomes; SF-36 = short-form 36-item health survey

Results were similar for correlations between PRO responses and DAS28 responses, with SF-36 PCS and MCS scores showing the lowest correlations with DAS28 responses (82.4% and 77.6%, respectively) and physical function, fatigue, pain and global assessment of disease activity showing the highest correlations (88.0%, 90.6%, 92.8% and 93.6%, respectively).

## Discussion

Over the past decade, the management of RA has changed dramatically as a result of the development of the TNF inhibitors, which have resulted in improved long-term outcomes. Initially, improved clinical responses achieved by the use of TNF inhibitors were the primary focus of clinicians. Now the RA burden as reported by patients has come to be recognized as a significant and treatable component of the disease. Utilized together, clinical and patient-reported outcomes reflect the spectrum of patients' disease and best reflect the overall effectiveness of TNF inhibitor therapy in RA.

Concepts that have traditionally been shown to be among the most important to RA patients are physical function, pain, and tiredness (fatigue). However, it is now well-documented that mental health disturbance is an important consequence of RA and a central component of the assessment of HRQoL. Therefore in RAPID 1, the concepts that were assessed as reported by the patients were HRQoL (including emotional, social and physical components), fatigue, physical function, arthritis pain, and global assessment of disease activity.

Patients in this trial (RAPID 1) had substantially diminished HRQoL at baseline compared with age- and gender-matched US population norms (particularly in Vitality, Role Emotional, Physical Function, Role Physical and Bodily Pain), as illustrated by the spydergram of SF-36 scores at baseline (Figure 2). The US population norms were used only as a benchmark to allow comparisons between RA patients with available general population data. Following treatment with either dose of CZP plus MTX, patients reported improvements in HRQoL, or 'multidimensional function', at first assessment (week 12), with large improvements in both the physical and mental domains of HRQoL that exceeded MCID. In particular, energy (Vitality) and emotional state (mental health) domain scores approached normative values of the general population in the US, and the significant improvement in SF-36 MCS scores observed with CZP plus MTX treatment in this trial, which approached levels of improvement in PCS scores, is novel among studies of TNF inhibitors in RA [31-35]. The negative psychological effects of RA are well documented; many patients with RA have mood and anxiety disorders [36], and it has been reported that 21% to 39% of patients with RA experience significant depressive symptoms [37,38], although treatment of these aspects of the disease is often suboptimal. The improvements in SF-36 MCS and mental health scores following CZP treatment are thus particularly relevant for patients because they may help them return to normal levels of emotional functioning.

Patients treated with CZP plus MTX also reported significant improvements in global assessment of disease activity and physical function, as well as relief of pain and fatigue, as early as week 1 of treatment. Although there was a small decrease in the percentage of patients reporting clinically meaningful reductions in fatigue from week 4 to week 52, this is likely because of protocol-mandated withdrawal at week 16 for failure to achieve ACR20 responses at weeks 12 and/or 14, as well as imputation of withdrawn subjects with the conservative approach of non-responders after week 16. Reductions in fatigue and all other PROs with CZP plus MTX remained highly significant throughout the trial, with approximately 50% of patients reporting clinically meaningful reductions by week 52. In general, the effect of CZP on the patient-reported outcomes described herein are comparable with those reported with other TNF inhibitors in clinical studies [31-35	,39] as well as in studies in clinical practice [19,32,34,40-43]. However, the rapid improvements in patient-reported outcomes in CZP-treated patients, particularly those reflecting improved mental health, which have not previously been observed, are a particularly interesting characteristic of this new anti-TNF.

Patient's daily burden of disease and their poor HRQoL directly limit their everyday activities as well as productivity at work and within the home with negative consequences for society. Results from the RAPID 1 trial have demonstrated that CZP treatment improves productivity at work and at home, including fewer number of days lost engaging in family, social and leisure activities [44]. Furthermore, a *post hoc *analysis demonstrated that these improvements in productivity were closely reflected by similar changes in pain, physical function and fatigue (unpublished observations).

The data presented in this paper demonstrate that the CZP-treated patients experience early improvements in all patient-reported outcomes over PBO. In addition, there have been data suggesting that rapid improvements in disease activity are associated with better long-term outcomes [45]. Patients have a greater chance of avoiding long-term disability, allowing them to regain their normal levels of physical, emotional and social participation and greatly improving overall HRQoL.

Previously reported results from the RAPID 1 trial have demonstrated that treatment with CZP plus MTX rapidly and significantly reduces disease activity (as assessed by DAS28 scores) and improves the clinical signs and symptoms of RA (as assessed by ACR20 response rates) [15]. The improvements in patient-reported outcomes following CZP treatment reported herein thus support and complement these previously reported clinical improvements. In addition, the analyses correlating responses as assessed by improvements in PROs with clinical (ACR and DAS) responses demonstrate that patient-reported outcomes correlate well with physician-reported, clinical indices. Not surprisingly, because the HAQ-DI and pain are components of the ACR20 response rate criteria, physical function and pain were highly correlated with ACR20 response rates. In contrast, changes in SF-36 PCS and MCS scores had the lowest correlation with ACR20 and DAS responses. These results illustrated that although clinical measures and PROs are generally well correlated, assessment of only clinical measures does not capture all aspects of RA and its impact on patients' physical and mental health. Assessment of both clinical (physician-reported) and patient-reported outcomes is thus necessary to fully elucidate treatment benefit.

## Conclusions

The patient-reported assessments implemented in the RAPID 1 clinical trial demonstrated that RA patients experience rapid and sustained improvements in HRQoL, fatigue, physical function, disease activity and arthritis pain. These improvements were statistically significant and clinically meaningful from the first post-baseline assessment through to the end of the study period at one year. These results demonstrate that the benefits of CZP extend beyond clinical efficacy endpoints into areas that are more relevant and meaningful for patients on a daily basis.

## Abbreviations

ANCOVA: analysis of covariance; CZP: certolizumab pegol; DAS: disease activity score; DMARD: disease modifying antirheumatic drug; FAS: fatigue assessment scale; HAQ-DI: health assessment questionnaire - disability index; HRQoL: health-related quality of life; ITT: intent to treat; LOCF: last observation carried forward; MCID: minimal clinically important difference; MCS: mental component summary; MTX: methotrexate; NRS: numeric rating scale; PBO: placebo; PCS: physical component summary; PRO: patient-reported outcomes; PtGA: patient's global assessment of disease activity; RA: rheumatoid arthritis; RAPID 1: RA PreventIon of Structural Damage 1; SF-36: short-form 36-item health survey; TNF: tumor necrosis factor; VAS: visual analog scale.

## Competing interests

VS has received consulting fees from UCB, Inc. PM, GB, RvV, BC, EK, and AK have received research grants and consulting fees from UCB, Inc. EN is a Life Sciences Business & Decision consultant working as consultant for UCB, Inc. GC is an employee of UCB, Inc.

## Authors' contributions

PM, GB, RvV, BC, EK and AK made substantial contributions to the data acquisition, analysis, and interpretation and reviewed and revised the article content. VS, GC and EN participated in the analysis and interpretation of the data, and VS and EN drafted the manuscript. All authors read and approved the final manuscript.
